# Plant pathogen responses to Late Pleistocene and Holocene climate change in the central Atacama Desert, Chile

**DOI:** 10.1038/s41598-018-35299-2

**Published:** 2018-11-21

**Authors:** Jamie R. Wood, Francisca P. Díaz, Claudio Latorre, Janet M. Wilmshurst, Olivia R. Burge, Rodrigo A. Gutiérrez

**Affiliations:** 1 Manaaki Whenua Landcare Research, PO Box 69040, Lincoln, 7640 New Zealand; 2 Departamento de Genética Molecular y Microbiología, Pontificia Universidad Católica de Chile, Avda. Libertador Bernardo O’Higgins 340, Santiago, Chile; 3 FONDAP Center for Genome Regulation & Millennium Institute for Integrative Biology (iBio), Santiago, Chile; 4 Departamento de Ecología, Pontificia Universidad Católica de Chile, Alameda, 340 Santiago Chile; 5 Institute of Ecology and Biodiversity (IEB), Las Palmeras, 3425 Ñuñoa, Santiago Chile; 6 School of Environment, The University of Auckland, Private Bag 92019, Auckland, 1142 New Zealand

## Abstract

Future climate change has the potential to alter the distribution and prevalence of plant pathogens, which may have significant implications for both agricultural crops and natural plant communities. However, there are few long-term datasets against which modelled predictions of pathogen responses to climate change can be tested. Here, we use 18S metabarcoding of 28 rodent middens (solidified deposits of rodent coprolites and nesting material) from the Central Atacama, spanning the last ca. 49 ka, to provide the first long-term late Quaternary record of change in plant pathogen communities in response to changing climate. Plant pathogen richness was significantly greater in middens deposited during the Central Andean Pluvial Event (CAPE); a period of increased precipitation between 17.5–8.5 ka. Moreover, the occurrence frequency of Pucciniaceae (rust fungi) was significantly greater during the CAPE, and the highest relative abundances for five additional potentially pathogenic taxa also occurred during this period. The results demonstrate the promising potential for ancient DNA analysis of late Quaternary samples to reveal insights into how plant pathogens responded to past climatic and environmental change, which could help predict how pathogens may responded to future change.

## Introduction

The potential for future climate change to alter the distribution and prevalence of plant pathogens is a topic that has received extensive attention in scientific literature^1–3^. Much work has focussed on the likely impacts for agricultural crops (for examples see^4–7^), yet there has also been significant interest in the increased risk for invasive pathogens to affect native vegetation communities^8–10^. Modelling, based on the climatic preferences of pathogenic species, currently underpins most predictions about the likely responses of plant pathogens to different climate change scenarios e.g.^4,6,11^. However, there is an increasing appreciation for the contribution that long-term records can make to testing modelled predictions about biological responses to climate change^12–14^. With few long-term datasets^15^ currently available for plant pathogens, and a recent acceleration in rates of environmental and biological change associated with climate change^16,17^, new approaches are urgently required to generate such records.

Herbaria collections have been recognised as one potential resource for studying temporal dynamics in plant pathogen populations^18,19^, but are limited to just the last few hundred years. Late Quaternary palaeoecological records, extending over the past several millenia, provide an alternative option for gaining a longer-term view. Such records have revealed clues about the responses of many different organisms during past large-scale environmental and climate change events e.g.^20–22^. However, plant pathogens are phylogeneticaly diverse (including viruses, bacteria and eukaryotes) and leave little evidence in terms of the proxies conventionally used in late Quaternary palaeoecology (e.g. microscopic plant and invertebrate remains)^23^. This means that they have previously received little attention in late Quaternary palaeoecological studies.

**Figure 1:**
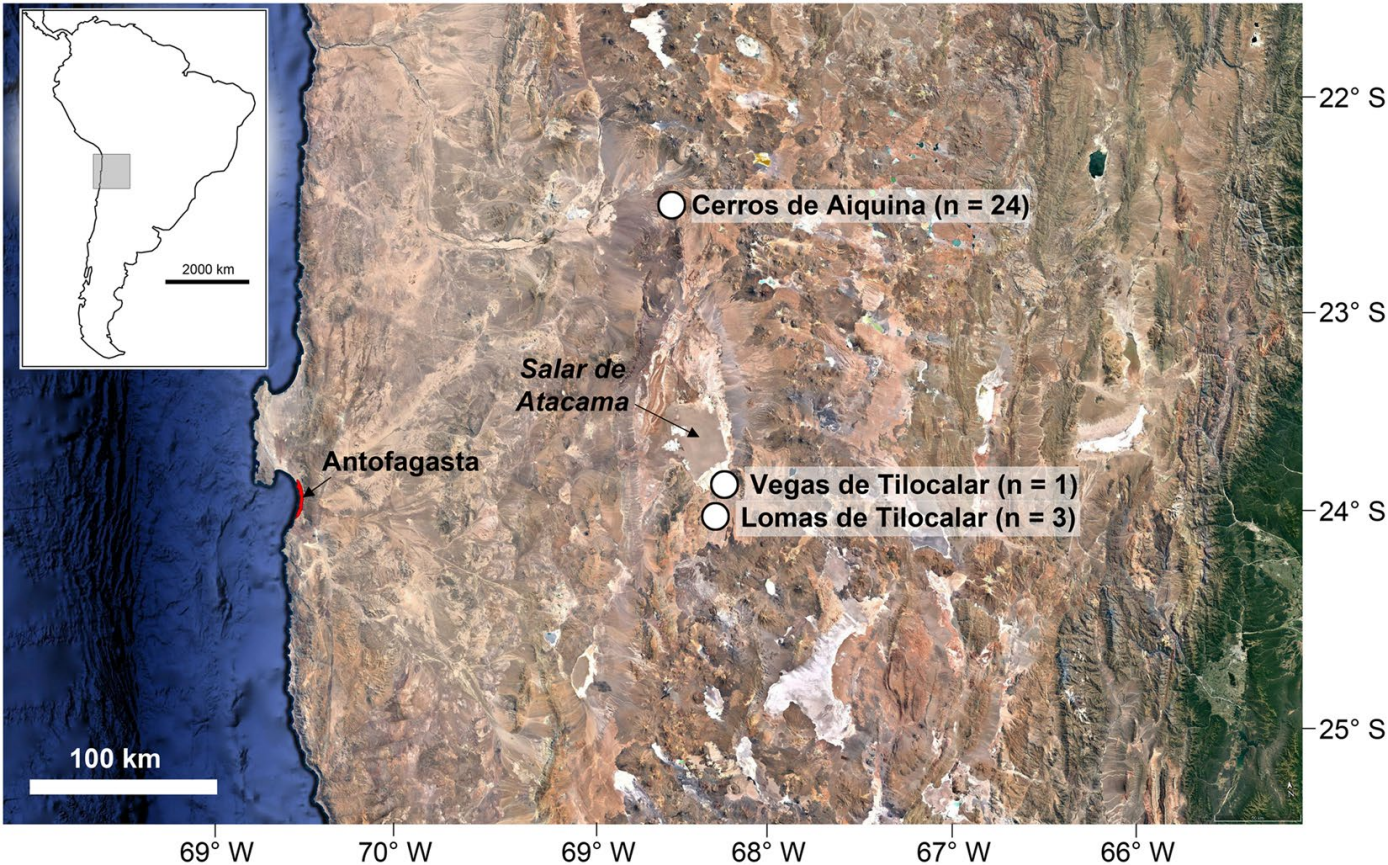
Collection localities of rodent middens used in this study. Numbers of middens sampled from each locality for ancient plant pathogen DNA are shown in brackets. Satellite imagery via Google Earth Pro 7.3.2.5491, data SIO, NOAA, U.S. Navy, NGA, GEBCO, image Landsat/Copernicus.

Ancient DNA analyses offer an alternative to conventional palaeoecological proxies, and are particularly useful for studying organisms that may leave no visible traces in sediments^24–26^, such as pathogens. Here, we use ancient DNA to provide the first long-term late Quaternary record of plant pathogens. Specifically, we use 18S metabarcoding to examine the prevalence and relative abundance of eukaryotic plant pathogenic taxa (fungi and oomycetes) in 28 fossil rodent middens from the central Atacama Desert (~22–24° S latitude, 68–69 W° longitude) (Fig. 1), spanning from 200–49,600 radiocarbon years in age. Rodent middens are concentrated deposits of biotic material, mostly vegetation remains and coprolites, cemented together by crystallized urine and typically represent a non-selective sample^27,28^ from the immediately local (<50 m radius) vegetation community^28^. Individual middens likely represent accumulation periods of months to decades, and therefore record brief snapshots of prehistoric ecosystems^29^. When deposited in dry crevices or caves rodent middens can be preserved for tens of thousands of years, making them an ideal resource for studying the effects of late Quaternary climate change on biological communities. Importantly, the temporal spread of our sampled rodent middens spans the Central Andean Pluvial Event (CAPE), a period of increased precipitation coinciding with the last glacial-interglacial transition, allowing us to test the response of plant pathogens to this major climate change event. For our purposes we treat the CAPE as a single event, although it actually consisted of two wet phases separated by a brief dry period. The wet phases are CAPE I (17.5–14.2 ka [calibrated years before present]), caused by a period of increased La Niña-like conditions in the central Pacific^27,30^, and CAPE II (13.8–8.5 ka)^28^, related to shifting moisture sources^27^. Most of the middens analysed here come from the upper margin of the Atacama’s Absolute Desert, a largely plant-less landscape where mean annual rainfall <10 mm/yr and a region where there has been major changes in plant community composition over the last 45,000 years^31^. Increases in precipitation during the CAPE (and possibly lower temperatures during CAPE I) drove many plant species 500 to 1000 m downslope, enriching local xerophytic communities especially with species from the Poaceae, Asteraceae and Solanaceae families^31,32^. Such climate-driven increases in local diversity may have also resulted in increases in the abundance of plant pathogens over time, a further hypothesis we explore here.

## Results

### Identity of plant pathogenic taxa

Short read lengths typically restrict taxonomic resolution and therefore limit the ability to make specific inferences about the ecology of identified taxa. However our analyses resolved six taxa within the middens that belong to groups that only include obligate plant pathogens. Three were oomycetes genera: *Albugo* (100% identity to *Albugo* spp., next nearest 94%), *Hyaloperonospora* (100–98% identity to *Hyaloperonospora*, next nearest 94%) and *Pythium* (100% identity to *Pythium* spp., other *Pythium* spp. 96% and lower), and three were fungal: Pucciniaceae, or rust fungi (100% identity only to taxa within this family), Ustilaginomycotina, or smut fungi (includes sequences with 100% identities to *Tilletia*, *Moniliella* and *Ustilago*) and *Fusarium culmorum* (100% identity, other *Fusarium* spp. have mismatches). Three additional fungal taxa identified from the middens include high proportions of plant pathogenic species: *Phytophthora* (98–96% identity to some *Phytophthora* spp., other *P*. spp. and genera lower), *Colletotrichum* (98% identity to *Colletotrichum*, next closest 95%) and Dothideomycetes (100% identities dominated by pathogenic taxa within Capnodiales, particularly Mycosphaerellaceae, and Botryosphaeriaceae). As we could not resolve any taxa to species-level we could not be unequivocal about the pathogenic status of the identified taxa. However, we refer to these nine taxa hereafter as plant pathogenic taxa with this caveat in mind. DNA sequences attributed to the nine taxa (Table 1; S1) were recovered from 18 (64.3%) of the middens.

**Table 1:** Plant pathogenic and potentially plant pathogenic taxa^#^ detected in rodent middens from the central Atacama.

Taxon/metric	N	Maximum value (% total reads^%^, n, or diversity index)	Age of max. abundance Cal. years BP	GLM		Welch t-test		
				statistic	*P*	t	df	*P*
**Fungi**								
*Colletotrichum* ^#^	2	0.0200^%^	15,194 (CAPE I)	−1.344	0.179	1.135	10	0.141
*Fusarium culmorum*	2	0.3583^%^	197	−0.32	0.749	−0.980	16	0.829
Pucciniaceae	9	0.5208^%^	15,194 (CAPE I)	−2.628	0.009*	2.213	10	0.026*
Ustilaginomycotina	2	0.0021^%^	12,752 (CAPE II)	−0.32	0.749	0.58	12.9	0.285
Dothideomycetes^#^	16	3.6057^%^	15,194 (CAPE I)	−0.557	0.577	0.645	16.7	0.264
**Oomycetes**								
*Albugo*	1	0.0518^%^	7,424	0.423	0.673	−1	16	0.834
*Hyaloperonospora*	6	0.7178^%^	3,492	−0.602	0.55	−1.064	16.3	0.849
*Phytophthora* ^#^	4	0.0388^%^	15,194 (CAPE I)	−1.9	0.057	1.475	10	0.086
*Pythium*	2	0.0070^%^	10,341 (CAPE II)	−0.32	0.749	−0.752	17.6	0.769
Total pathogen reads	18	4.1854^%^	15,194 (CAPE I)			0.676	17.2	0.508
Pathogen taxon richness	18	6	10,341 (CAPE II)			1.971	13.8	0.035*
Shannon Diversity	18	1.079	7, 532			1.404	23.9	0.087

N refers to the number of midden samples in which the taxon/metric was detected.

### Responses to climate

**Figure 2:**
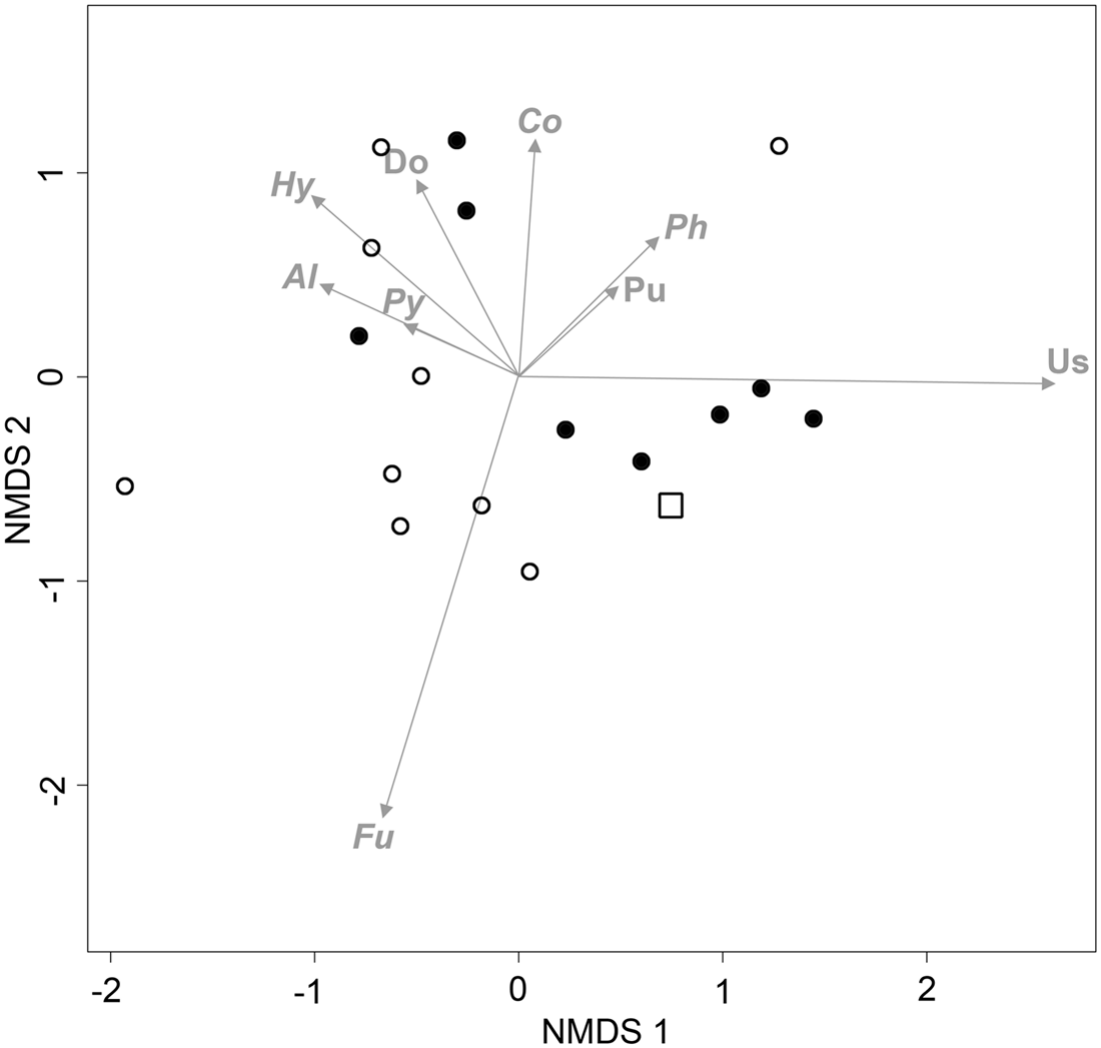
MDS plot of rodent middens based on plant pathogen assemblages detected using 18S metabarcoding. Filled circles represent middens deposited during CAPE, empty circles represent middens deposited outside CAPE, and the square represents the oldest midden sampled (49,600 yrs BP). Al, *Albugo*; Co, *Colletotrichum*; Do, Dothideomycetes; Fu, *Fusarium culmorum*; Hy, *Hyaloperonospora*; Ph, *Phytophthora*; Pu, Pucciniaceae; Py, *Pythium*; Us, Ustilaginomycotina.

**Figure 3:**
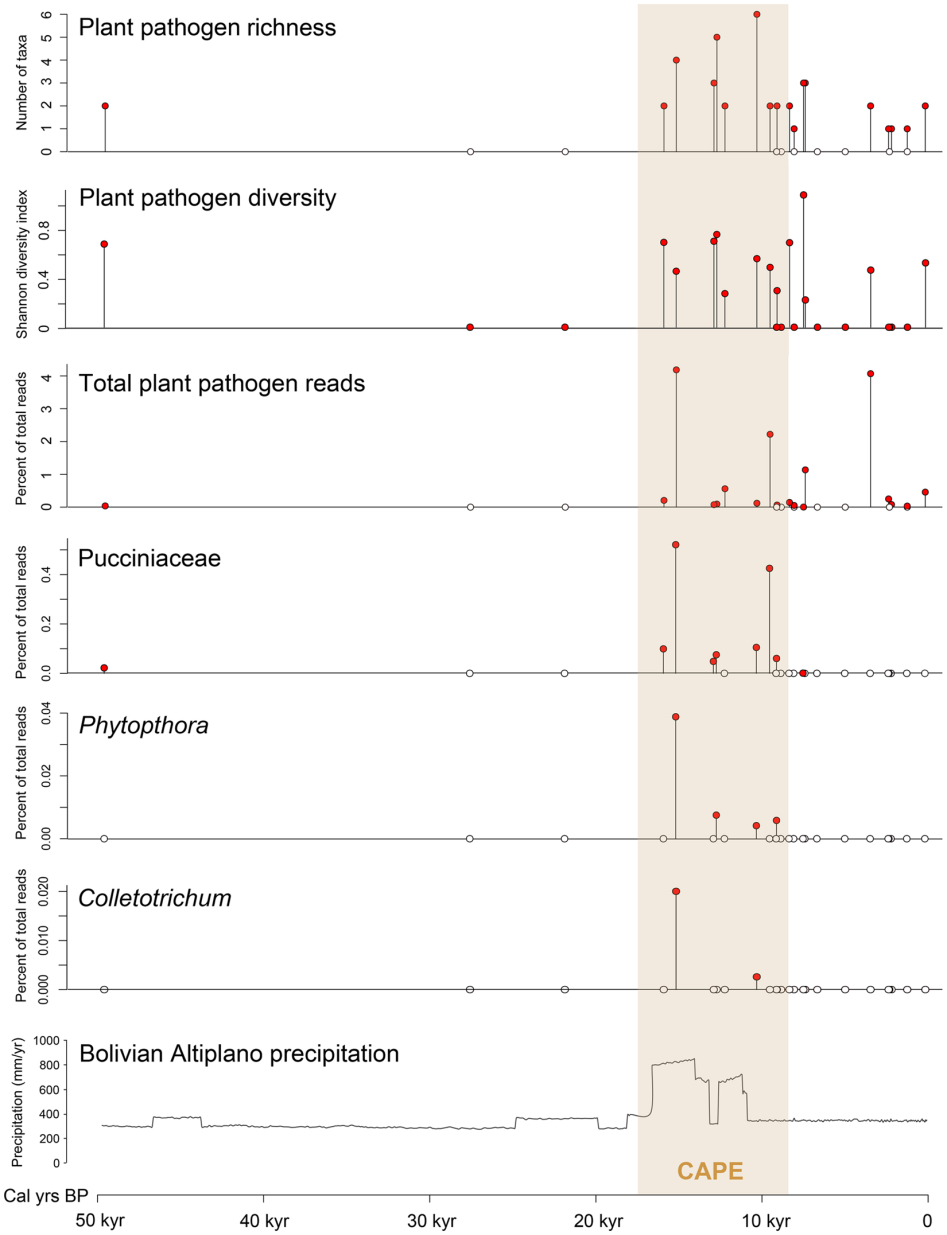
Plant pathogens detected using ancient DNA in rodent middens from the central Atacama. Empty circles represent midden samples where the plant pathogen taxon was not detected. The data are shown in relation to the local timing of the Central Andean Pluvial Event (CAPE)^28^ and alongside a precipitation curve for the Bolivian Altiplano^53^ ~600 km to the north of the study area.

There appears to be no clear distinction of CAPE and non-CAPE middens based on the composition of the plant pathogen assemblages identified by our metabarcoding primers (Fig. 2). However, there was clearer discrimination between CAPE and non-CAPE middens for other assemblage-level metrics. For example, the highest richness of plant pathogenic taxa and highest total relative abundance of plant pathogenic reads were observed in midden samples deposited during the CAPE (Fig. 3; Table 1), and plant pathogen richness was significantly greater in CAPE middens than non-CAPE middens (Table 1).

For individual plant pathogenic taxa, distinct patterns in occurrence and abundance were observed that corresponded to the CAPE. The highest relative abundances for six of the nine taxa (*Colletotrichum*, Pucciniaceae, Ustilaginomycotina, Dothideomycetes, *Phytophthora* and *Pythium*) were recorded in middens deposited during the CAPE (Table 1). Pucciniaceae was significantly more abundant in middens deposited during the CAPE and occurred more frequently in middens deposited during the CAPE compared to those deposited earlier or later than the CAPE (Table 1; Fig. 3). *Phytophthora* and *Colletotrichum* were only detected in middens deposited during the CAPE (Table 1; Fig. 3).

### Plant community effects

**Figure 4:**
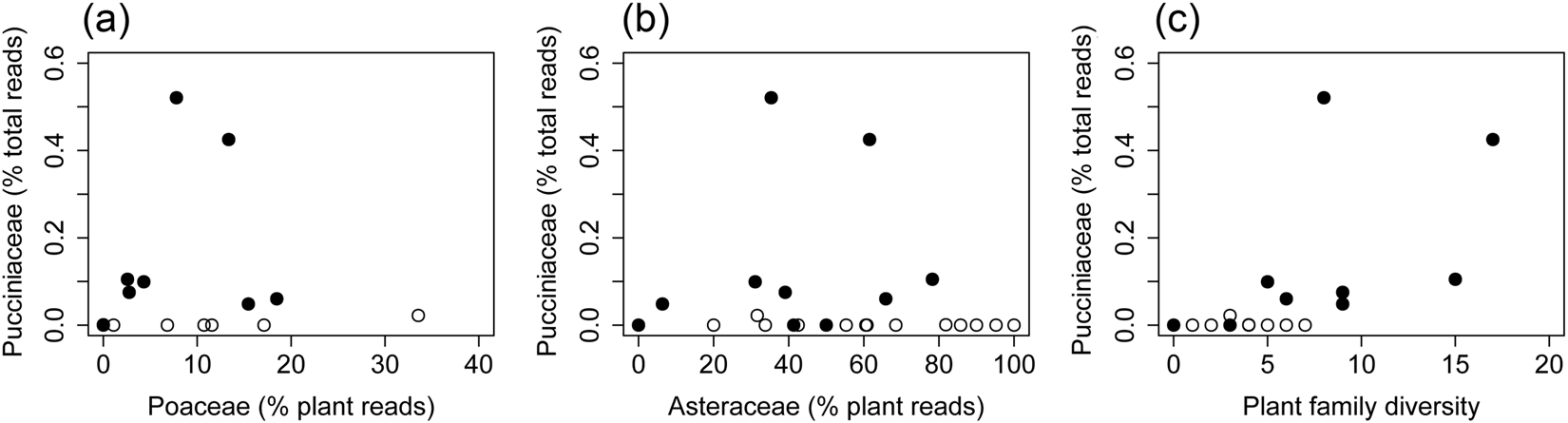
Relationships between the relative read abundance of Pucciniaceae and Poaceae, Asteraceae and plant family diversity, in rodent middens from the central Atacama. Middens deposited during the Central Andean Pluvial Event (CAPE) are represented by filled circles, while those deposited earlier or later than CAPE are represented by empty circles.

Although Pucciniaceae infect hosts from most orders of Angiosperms, representatives of the predominantly herbaceous families Asteraceae and Poaceae are known to be more commonly affected^33^. As our metabarcoding primers also amplified plant DNA, we were able to examine the correlation between the relative abundances of Pucciniaceae vs. Poaceae and Asteraceae reads in the midden samples (Fig. 4). The overall linear correlation of the relative abundance of Pucciniaceae vs Poaceae and Asteraceae reads was very weak and not significant (Fig. 2) (R^2^ = 0.05, p = 0.251 and R^2^ = < 0.01, p = 0.97 respectively), and the strength of the positive relationships only marginally increased for samples deposited during CAPE (Fig. 4) (R^2^ = 0.14, p = 0.253 and R^2^ = 0.08, p = 0.399 respectively). Both Poaceae and Asteraceae reads were obtained at relatively high abundances in several non-CAPE middens, yet Pucciniaceae reads were rare in the same samples (Fig. 4). The relative abundance of Pucciniaceae reads also increased with increasing plant diversity (at family-level) (R^2^ = 0.39, p = 0.06) (Fig. 2), yet higher plant diversity was also observed during CAPE (Fig. 4).

## Discussion

The plant pathogenic taxa detected in the middens displayed a range of different responses during the CAPE. Some taxa exhibited no significant difference in occurrence frequency or abundance between middens deposited during or outside the pluvial events, while others appeared to increase in abundance and/or occur at higher frequencies during the CAPE. These varying responses indicate that different taxa were responding to different climatic or biological drivers. Pucciniaceae appeared to be the taxon most influenced by the CAPE, exhibiting significant increases in both frequency of occurrence and relative abundance in middens deposited during the CAPE (Table 1). We suggest that there are three potential explanations for the response of this taxon. First, an increase in local plant diversity during the pluvial event may have resulted in an increase in the abundance of preferential host species. Second, the actual prevalence of the pathogenic taxon may have increased directly as a response to a change to more favourable climatic conditions. Third, an increase in plant density during the pluvial event may have driven a landscape-level increase in local plant pathogen abundance.

Higher plant diversity observed during pluvial events (Fig. 4) indicates that the first explanation may have some credence. Many genera within the Pucciniacae are known to occur widely on grasses (e.g. *Puccinia*, *Uromyces*), and fossil pollen work has demonstrated a regional increase in grasses in these middens during the Atacama pluvial event^29,34^, with plant macrofossil studies showing major increases in grass diversity over the same period^31^. However, the patterns demonstrated in our data show that there was no (or very weak) correlation of the relative abundance of Poaceae/Asteraceae and Pucciniaceae reads in our samples. Furthermore, abundant DNA reads of Poaceae/Asteraceae were present in several middens that were either younger or older than the CAPE, yet Pucciniaceae was rare in these samples (Fig. 4). Together, these data indicate stronger support for the second explanation, i.e. that climate had a direct effect on Pucciniaceae abundance, and not just an indirect effect via changes in the plant community composition. Presumably this direct effect was driven by increased moisture availability, as Pucciniaceae^35–37^ species are known to benefit from increases in precipitation frequency and/or humidity (e.g. through increased survival of mycelia in plant tissues, increased spore release and higher atmospheric spore concentrations). These kinds of favourable climatic conditions prevailed in the Atacama Desert during the CAPE, when Pucciniaceae read abundance was correlated with the abundance of preferred hosts. However, during less favourable drier periods Pucciniaceae was rare, irrespective of the abundance of preferred hosts. Soil DNA evidence suggests that Puccinaceae are present but very rare in the driest parts of the Atacama Desert today^38^.

Despite the evidence for direct climatic influences, density-dependent effects (the third explanation) are difficult to rule out, as the selective and highly localized collection and concentration of plant macrofossils in rodent middens makes the actual density of local vegetation communities difficult to resolve. Pollen analyses of middens more generally reflect the local and regional vegetation at the elevation of the midden and have been suggested to provide a more accurate reconstruction of plant density surrounding the midden sites^28^ and therefore may provide a tool for examining density-dependent effects. However, we found no clear correlation between pathogen abundance and total pollen concentration, nor between Pucciniaceae abundance and Poaceae/Asteraceae pollen concentration (Fig. S1). This is indicative that the density of plants on the landscape did not play an important role in determining pathogen abundance and lends more support to climate being the main driver.

While Pucciniaceae appears to have been significantly affected by CAPE in the central Atacama Desert, other putative plant pathogenic taxa were not affected to the same extent. Although maximum relative abundances for five of these taxa occurred in middens deposited during CAPE, there were no significant differences in the abundance or occurrence frequency of these taxa between CAPE and non-CAPE middens (Table 1). There may be a range of explanations for this pattern. One possibility is that that the specific environmental or climatic factors driving changes in abundance and distribution may differ between taxa, and some of these drivers (e.g. mean temperature) might have not changed as markedly during CAPE as others (e.g. precipitation). Host-jumping e.g.^39–41^ is another process that could affect an observed pattern of plant pathogen abundance over long time periods and potentially mask climatic influences.

Although pathogen DNA has previously been sequenced from historic (<200 year old) plant tissues^15,42–44^, our study reports the oldest DNA sequences so far recovered for plant pathogens. None of the plant pathogenic taxa detected in the middens were particularly common (all except Dothideomycetes occurred at <1% of total reads per midden), emphasising the importance of high-throughput sequencing for detecting and studying these organisms in environmental samples. Further insights, particularly around confirming the pathogenic status of present taxa, could be gained by the application of taxon-specific barcoding primers that can utilise more complete reference sequence databases and provide improved taxonomic resolution (e.g. ITS for fungi, 16S for bacteria). Moreover, shotgun sequencing could also provide information on viral pathogens within such samples^45^.

Even when a large body of data on the environmental tolerances of a particular plant pathogenic taxon exists, predictions about how prevalence might be affected by future global change can remain elusive^37^. The results of this study demonstrate the excellent potential for ancient DNA analysis of late Quaternary samples to reveal insights into pathogen responses to past climatic and environmental change, at least on a regional scale, which may provide baselines for predicting responses to future change.

## Materials and Methods

### Rodent middens

Twenty-eight rodent middens from three discrete localities bordering the Salar de Atacama basin (2300 m above sea level) in the Antofagasta region of the central Atacama Desert, northern Chile (Fig. 1; Table S2), were used in this study. Twenty-four middens ranging from 200 to 49,600 radiocarbon years in age were analysed from Cerros de Aiquina (~22° 21′ S, 68° 18′ W) (2967–3355 m above sea level). Three middens ranging from 3,305 to 18,100 radiocarbon years in age were analysed from Lomas de Tilocalar (~23° 55′ S, 68° 10′ W) (2872–2893 m above sea level). A single midden dating to 23,430 radiocarbon years was analysed from Vegas de Tilocalar (~23° 44′ S, 68° 6′ W) (2414 m above sea elevel). Radiocarbon dates for the middens were calibrated using ShCal13^46^ in OxCal v.4.3.2^47^ and median ages were used for analyses. Based on recent age estimates for the CAPE in the central Atacama^28^, three of the sampled middens were older than CAPE, two were deposited during CAPE I, nine were deposited during CAPE II, and 14 were younger than CAPE. Further details of middens and radiocarbon ages are provided in Supplementary Table 1.

### Molecular analyses

#### DNA extraction and metabarcoding

DNA extraction and PCR setup were performed in a dedicated ancient DNA laboratory at Landcare Research, Lincoln, New Zealand. DNA was extracted from subsamples (3.45–6.03 g, mean = 5.19 g) of each midden using the MoBio PowerMax kit. DNA extractions were performed in four batches, each consisting of seven samples and one negative extraction control. A variable length region (40–120 bp within eukaryotes) of the 18S rRNA gene was amplified using the primers Nem18SF and Nem18SR^48^ with linker sequences at the 5′ end. A detailed assessment of the taxonomic biases of these primers has yet to be performed, but alignment of the primers with 18S sequences from plant pathogens (Fig. S2) and parasitic eukaryotes^48^ show that any mismatches are concentrated at the 5′ ends. Moreover, sequencing results that we have obtained using these primers indicate that they are highly specific to eukaryotes and amplify DNA from a broad range of eukaryote taxa. The short amplicon length makes them ideal for ancient DNA, and they target a region that is variable enough to provide useful taxonomic resolution. PCR reactions (12.5 μL) contained 1 μg/mL BSA, 1x PCR buffer, 2 mM MgSO_4_, 40 μM dNTPs, 0.4 μM each primer, 1U Platinum HiFi Taq and 1 μL DNA extract. Cycling conditions were an initial denaturation at 94 °C for 3 min, followed by 55 cycles of 94 °C for 30 sec, 55 °C for 30 sec, 68 °C for 45 sec and a final elongation at 68 °C for 10 min. Each PCR was repeated in triplicate and PCR batches included negative controls. Products were visialised on 3.5% agarose gels. None of the extraction or PCR negative controls produced visible bands of amplified product on agarose gels. Following this, 1 μL of each amplicon library was dual indexed using 0.75 μL of 2 μM indexed adapters (P5/P7) in 20 μL reactions using iTaq DNA polymerase and following the manufacturers reaction mix. Cycling conditions were 94 °C for 2 min, followed by 7 cycles of 94 °C for 20 sec, 55 °C for 10 sec, 72 °C for 30 sec and a final elongation at 72 °C for 10 min. Indexed libraries were quantified using a Qubit 2.0. Equal amounts were pooled and purified using a Pippen Prep with a size-selection window of 220–260 bp. After final quantification and pooling, sequencing was performed using 2 × 150 bp kit on an Illumina MiSeq (Macrogen, South Korea).

#### Bioinformatics

For read processing, the following commands were implemented in USEARCH^49^: (1) paired-end reads were assembled using –fastq_mergepairs; (2) reads with a maxee >1 were discarded using–fastq_filter; (3) primers were removed using –fastx_truncate; (4) dereplication was performed using–fastx_uniques; (5) error correction, chimera removal and exclusion of singletons was performed using –unoise^50^. Processed reads were aligned with the NCBI nucleotide database using BLAST and the nearest 50 matches to each were recorded. Taxonomic assignment was performed via MEGAN v.6.9.1^51^ using a minimum LCA score of 50.0 and parsing to matches within 5% of the top score. Comparison of OTUs derived from individual PCRs demonstrated a good level of reproducibility (accounting for differences in sequencing depth), and in all cases the most abundant OTU in each of the triplicate PCRs was the same and the relative abundance of OTUs was reasonably consistent (Fig. S3). On this basis, results from the triplicate libraries for each midden were combined. As a further quality control step, sequences attributed to plant pathogenic taxa were again aligned against the NCBI nucleotide database using BLAST, this time returning the nearest 500 matches. These were examined manually to assess whether the taxonomic assignments provided by MEGAN remained robust alongside the higher number of nearest matches (allowing us to explore: 1) saturation of the top 50 matches by taxa with high numbers of GenBank sequences; and 2) high % identity scores outside the nearest 50 matches).

### Statistics

Differences in the likelihood of occurence of pathogenic taxa during and outside the CAPE were tested using a generalised linear model with a binomial error structure and logit link; where data separation occurred (*Albugo*, *Phytophthora*, *Colletotrichum*) the mean bias-reduced fit^52^ was implemented using the brglm2 package in R. Models were run for each taxa separately. Differences in the mean relative abundance were tested using Welch’s two sample t-test (one-sided), which is robust to unequal variances. Mean abundance was quantified relative to total DNA reads rather than total plant pathogen reads. In all cases we tested for a difference between CAPE (17.5–8.5 ka) and non-CAPE periods (<8.5 ka and >17.5 ka). Analyses were repeated with the 49,600 yr old midden excluded (due to it being a temporal outlier compared with other middens) but no differences were observed.

## Electronic supplementary material

Supplementary information

## Data Availability

Unprocessed reads from each midden sample (.fastq) will be made available via Landcare Research’s Datastore, https://datastore.landcareresearch.co.nz/.
