# Supplementary material for: Plant pathogen responses to Late Pleistocene and Holocene climate change in the central Atacama Desert, Chile
Source: Sci Rep. 2018 Nov 21;8:17208. doi: 10.1038/s41598-018-35299-2 (PMC6249261; doi:10.1038/s41598-018-35299-2)
Supplement: Supplementary file 1 — Supplementary information [file 41598_2018_35299_MOESM1_ESM.pdf]

SUPPLEMENTARY INFORMATION

Plant pathogen responses to Late Pleistocene and Holocene climate change in the central Atacama Desert, Chile

Jamie R. Wood, Francisca P. Díaz, Claudio Latorre, Janet M. Wilmshurst, Olivia R. Burge, and Rodrigo A. Gutiérrez

Fig. S1 | Correlation of pollen concentrations with relative abundance of pathogen DNA reads. Middens deposited during the Central Andean Pluvial Event (CAPE) are represented by filled circles, while those deposited earlier or later than CAPE are represented by empty circles. Pollen concentration data was available only for middens with code prefix CDA (see Table S1).

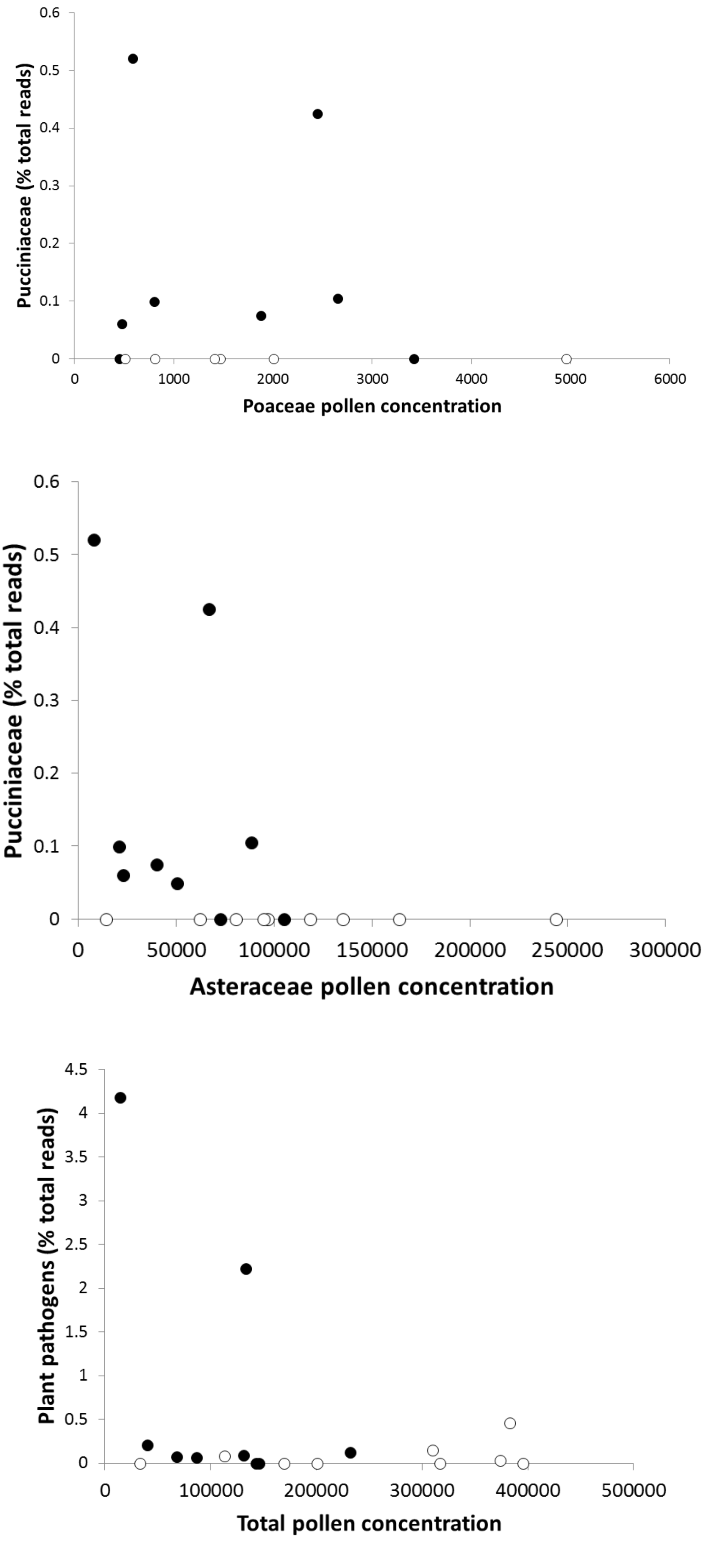

Fig. S2 | Alignment of Nem18S primers with representative 18S sequences from plant pathogenic and putative pathogenic taxa detected in middens. GenBank accession numbers are given for the reference sequences. *Puccinia lagenophorae* represents Pucciniaceae, *Tilletia goloskokovii* represents Ustilaginomycotina and *Phyllosticta gardeniicola* represents Dothideomycetes/

```
Consensus                                  ATTCCGATAACGAACGAGACCTTCRCCTGCTAAATAGTCCSGCTTAC----TTTGGTAGGTTG-CTGACTTCTTAGAGGGACTATYGG

Nem18SF/18SR binding sites                 ....................                                               ...............M.G...

JN635227 Phytophthora capitosa             ......T...............C...G......C....TT.......CATT...........A-GG................T....

HQ643854 Pythium takayamanum               ......T...............C...G...........TT.......AATT...T........-GA................T....

AY125413 Puccinia lagenophorae             .A.T....................T...............A...GG.----.A...CT..C..-.....................AA

KF853245 Albugo candida                    ......T...............C...A.-.........TT.......A--T...T.......TTGA................T....

AY742752 Hyaloperonospora parasitica       ......T...............C...G......C....T...T....CATT.........CGTGTG................T....

DQ832247 Tilletia goloskokovii             .......................A.........C...C.....CG..----......TA.C..-...G....................

MH020175 Phyllosticta gardeniicola         ...G...................A.............C.....CCG.----.....CG...C.-.C.G....................

KM085967 Colletotrichum gloeosporoides     ...G...................A.............C...TA..GT----.....C..TAC.-...G....................

DQ133022 Fusarium culmorum                 ...G...................A.............C...TA..G.----.C...C..TAC.-...G....................
```

Fig. S3 | Reproducibility plots for three separate PCRs sequenced for each midden sample. Each plot ranks the 50 most abundant OTUs from the PCR that yeilded the greatest total number of reads (red). The abundance of the same OTUs from the other two PCRs are shown by the blue and black curves (black represents the PCR that yeilded the lowest total number of reads). Gaps represent where a particular OTU was not detected in a PCR. The plots include all OTUs amplified, not just those assigned to plant pathogens. The blue and black curves all follow the same trends in comparison to the red curves (i.e. the relative abundance of OTUs detected in each of the three replicates is consistent within each replicate). The key differences between replicates are missing OTUs (rather than differences in relative abundance), and this appears to increase with decreasing sequencing depth.

AD121 (197 BP)

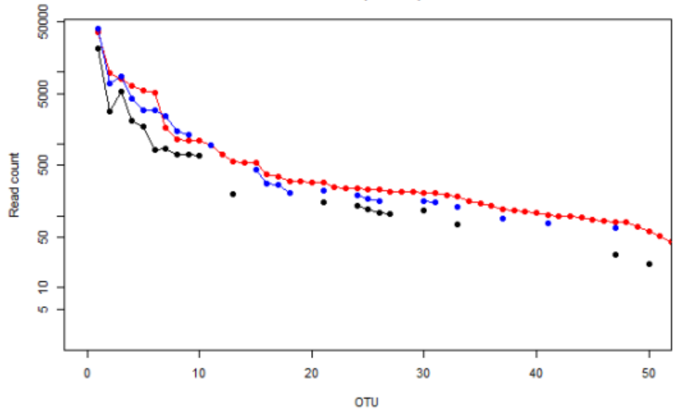

AD113 (1,284 BP)

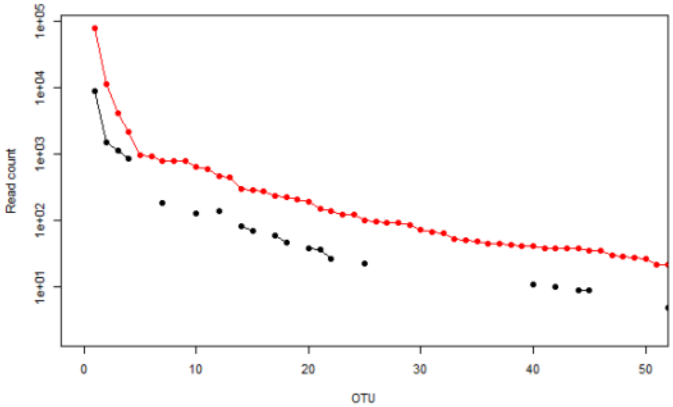

AD137 (1,286 BP)

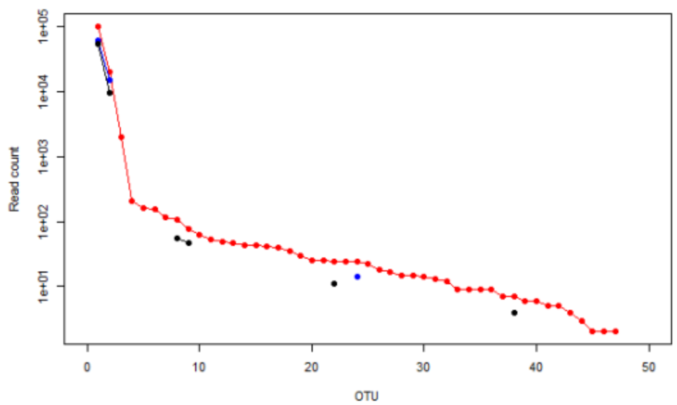

AD111 (2,231 BP)

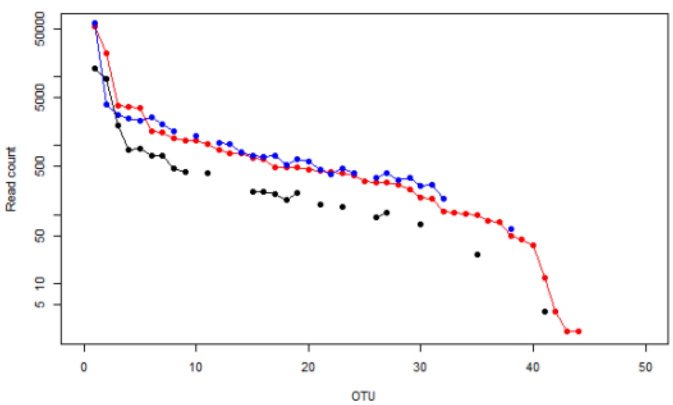

AD112 (2,358 BP)

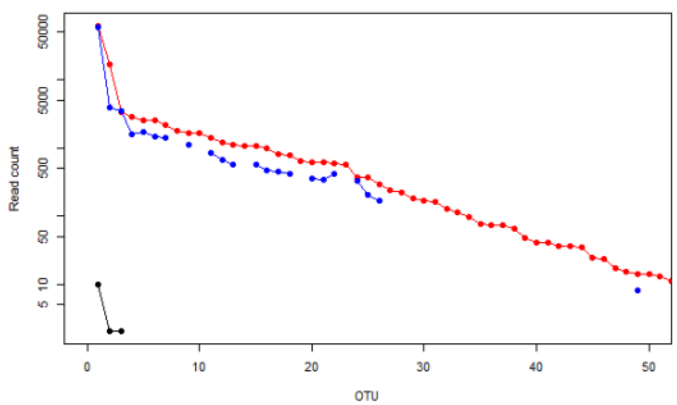

AD129 (2,406 BP)

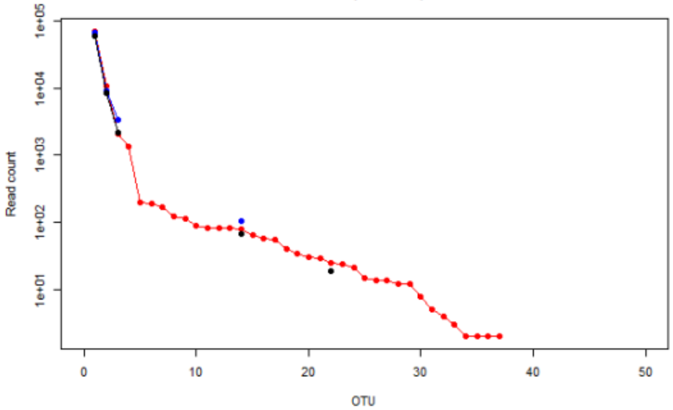

AD141 (3,492 BP)

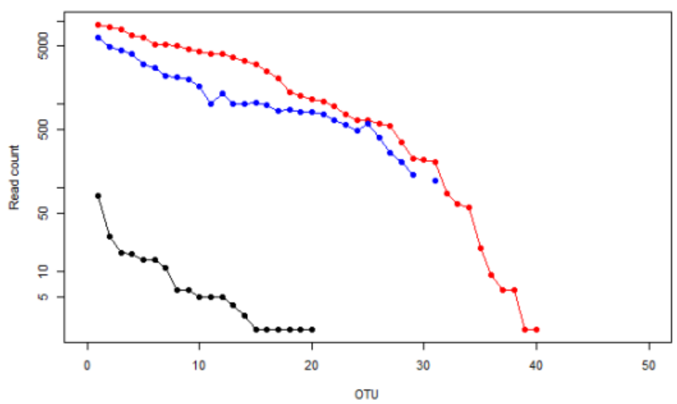

AD115 (5,020 BP)

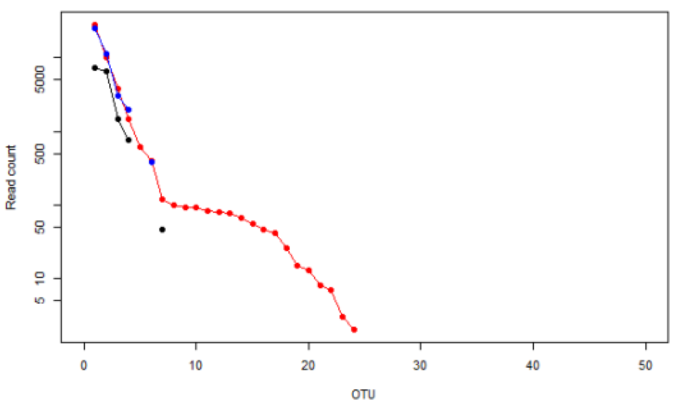

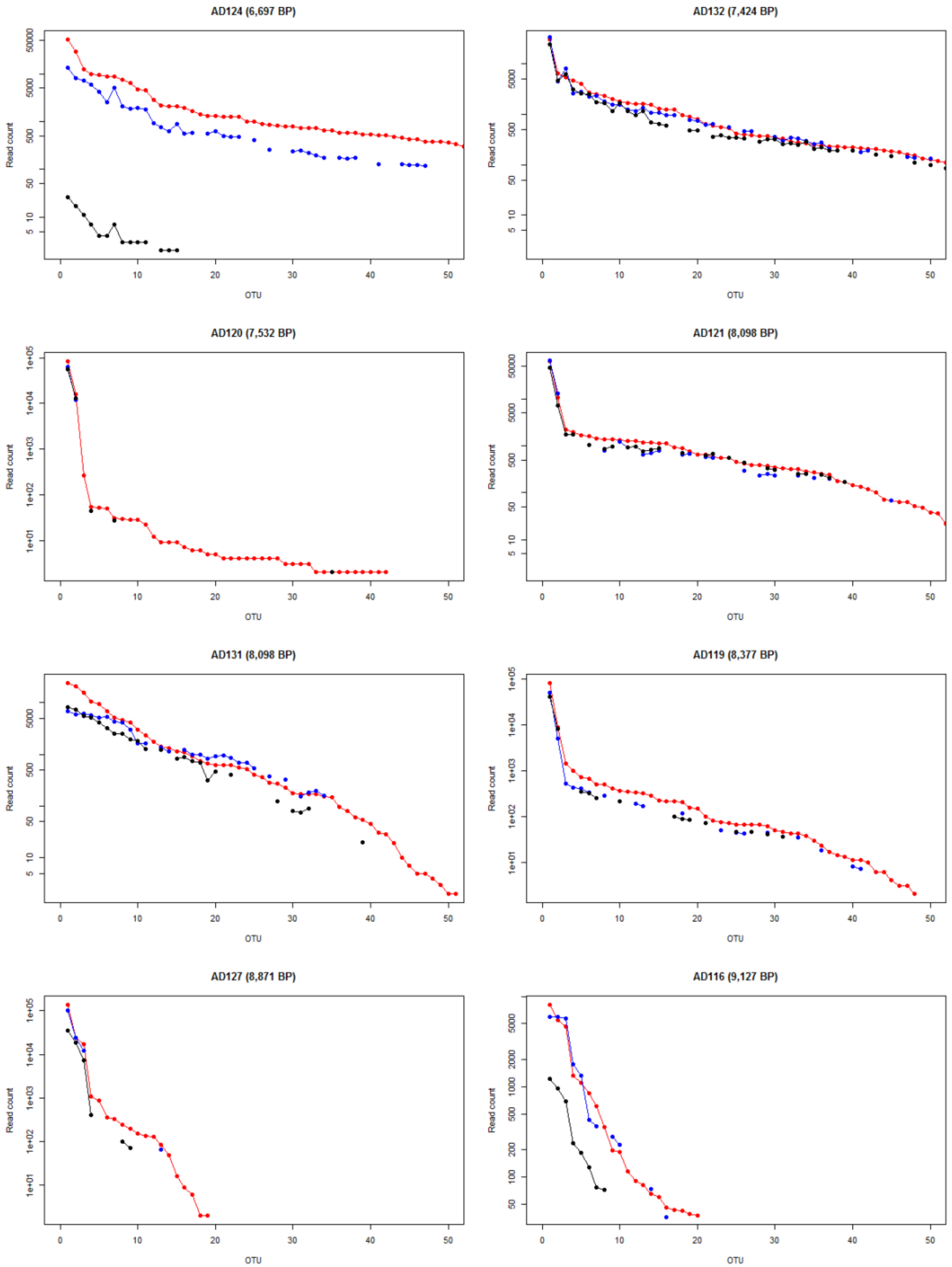
AD124 (6,697 BP)
AD132 (7,424 BP)
AD120 (7,532 BP)
AD121 (8,098 BP)
AD131 (8,098 BP)
AD119 (8,377 BP)
AD127 (8,871 BP)
AD116 (9,127 BP)
Read count
OTU

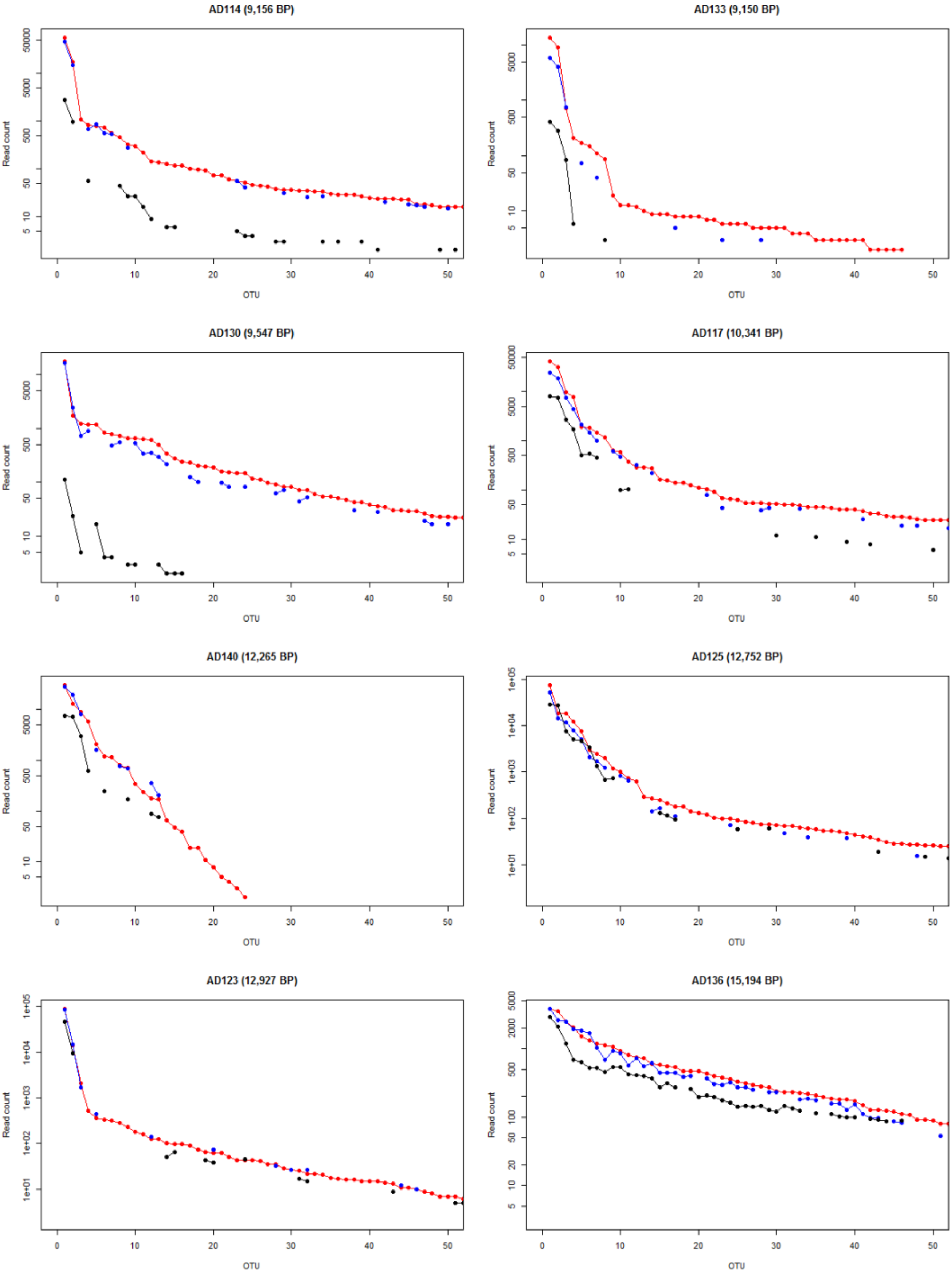
AD114 (9,156 BP)
AD133 (9,150 BP)
AD130 (9,547 BP)
AD117 (10,341 BP)
AD140 (12,265 BP)
AD125 (12,752 BP)
AD123 (12,927 BP)
AD136 (15,194 BP)
Read count
OTU

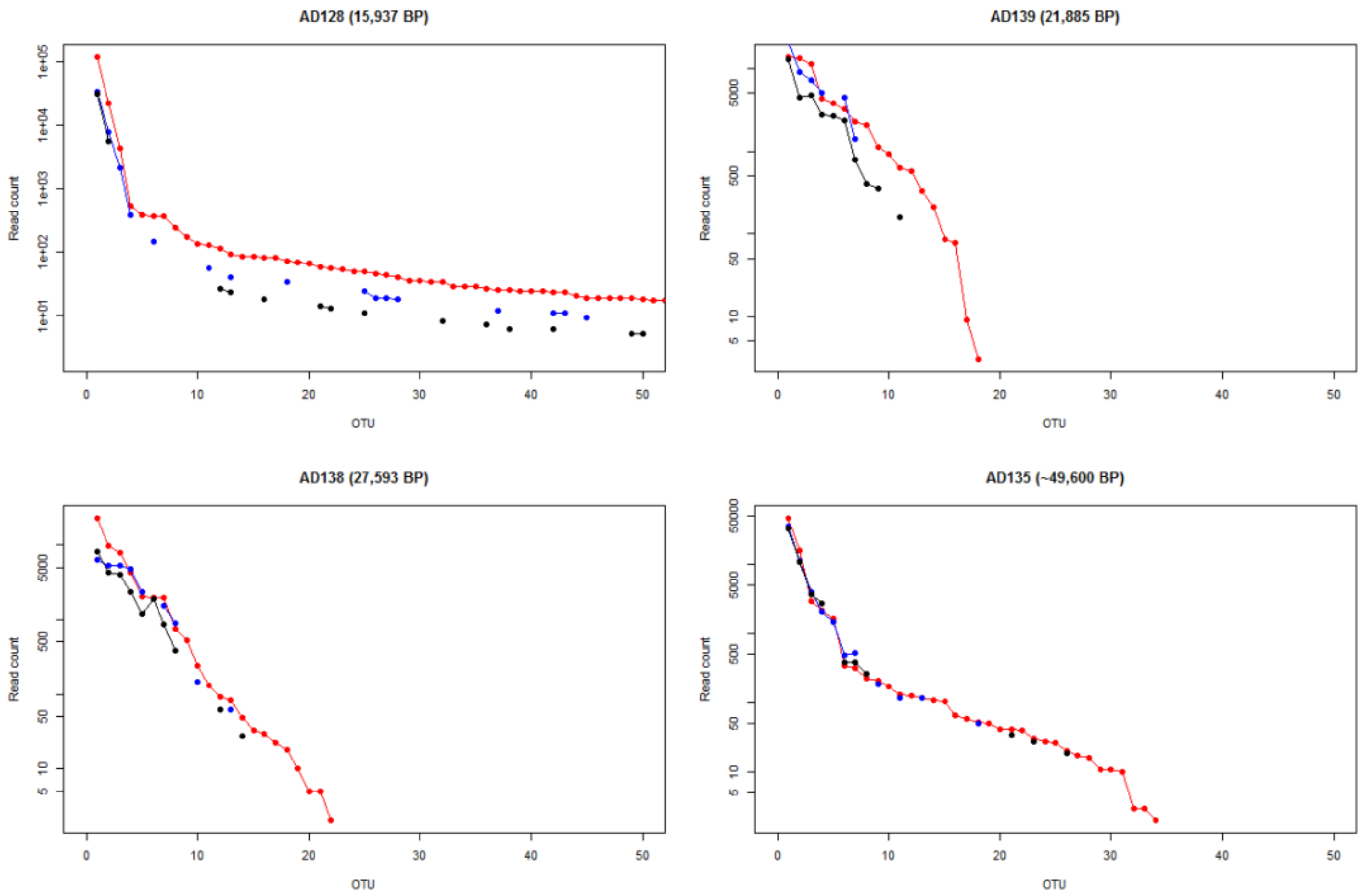
AD128 (15,937 BP)
AD139 (21,885 BP)
AD138 (27,593 BP)
AD135 (~49,600 BP)
Read count
OTU

Supplementary Table 1. Total read counts and read counts for plant pathogenic and potentially plant pathogenic taxa detected in rodent middens from the central Atacama.

Sample/PCR replicate	AD111A	AD111B	AD111C	AD112A	AD112B	AD112C	AD113A	AD113B	AD113C	AD114A	AD114B	AD114C
Median cal. age yrs BP	2231	2231	2231	2358	2358	2358	1284	1284	1284	9156	9156	9156
Total reads	97317	103627	32385	85131	14	112045	16077	792	106932	4818	68840	80593
Albugo												
Hyaloperonospora												
Pythium												
Pucciniaceae										6	58	29
Ustilaginomycotina												
Fusarium culmorum												
Phytophthora											2	7
Colletotrichum												
Dothideomycetes	588	1526	16	3					2	11	76	142

Sample/PCR replicate	AD115A	AD115B	AD115C	AD116A	AD116B	AD116C	AD117A	AD117B	AD117C	AD119A	AD119B	AD119C
Median cal. age yrs BP	5020	5020	5020	9127	9127	9127	10341	10341	10341	8377	8377	8377
Total reads	16665	45148	42949	3743	23242	23426	67046	101965	23400	61023	54595	100991
Albugo												
Hyaloperonospora									3	45	99	
Pythium							2					
Pucciniaceae							99	53	50			
Ustilaginomycotina												
Fusarium culmorum												
Phytophthora							8					
Colletotrichum									5			
Dothideomycetes							41	51	6	86	138	

Sample/PCR replicate	AD120A	AD120B	AD120C	AD121A	AD121B	AD121C	AD122A	AD122B	AD122C	AD123A	AD123B	AD123C
Median cal. age yrs BP	7532	7532	7532	8098	8098	8098	197	197	197	12927	12927	12927
Total reads	69553	74546	100512	79477	105164	100052	85134	77428	41162	60234	109559	107099
Albugo												
Hyaloperonospora										2		
Pythium												
Pucciniaceae										26	65	43
Ustilaginomycotina			3									
Fusarium culmorum							243	205	282			
Phytophthora												
Colletotrichum												
Dothideomycetes			2	963	524	1900	439	10	2	26	39	86

Sample/PCR replicate	AD124A	AD124B	AD124C	AD125A	AD125B	AD125C	AD127A	AD127B	AD127C	AD128A	AD128B	AD128C
Median cal. age yrs BP	6697	6697	6697	12752	12752	12752	8871	8871	8871	15937	15937	15937
Total reads	68341	197233	94	84798	145917	102094	62184	182427	141775	47618	149415	38857
Albugo												
Hyaloperonospora												
Pythium												
Pucciniaceae				68	104	78				137	79	18
Ustilaginomycotina						7						
Fusarium culmorum						15						
Phytophthora				12	9	4						
Colletotrichum												
Dothideomycetes				164	56	153	24			287	366	69

Sample/PCR replicate	AD129A	AD129B	AD129C	AD130A	AD130B	AD130C	AD131A	AD131B	AD131C	AD132A	AD132B	AD132C
Median cal. age yrs BP	2406	2406	2406	9547	9547	9547	8098	8098	8098	7424	7424	7424
Total reads	84780	85544	73624	32754	190	27035	71354	120016	52918	86033	78955	62952
Albugo										118		
Hyaloperonospora							72		53			
Pythium												16
Pucciniaceae				159		96						
Ustilaginomycotina												
Fusarium culmorum												
Phytophthora												
Colletotrichum												
Dothideomycetes	169	218	327	783	2	317	151	561		1621	37	1124

Sample/PCR replicate	AD133A	AD133B	AD133C	AD135A	AD135B	AD135C	AD136A	AD136B	AD136C	AD137A	AD137B	AD137C
Median cal. age yrs BP	9150	9150	9150	49600	49600	49600	15194	15194	15194	1286	1286	1286
Total reads	24349	813	11023	56115	70718	59059	18078	29408	32387	77610	64335	125651
Albugo												
Hyaloperonospora												
Pythium												
Pucciniaceae					41		62	232				
Ustilaginomycotina							180	930	311			
Fusarium culmorum												
Phytophthora							31					
Colletotrichum							16					
Dothideomycetes	13	2	5	214	20	132	733	867	1506	45		43

Sample/PCR replicate	AD138A	AD138B	AD138C	AD139A	AD139B	AD139C	AD140A	AD140B	AD140C	AD141A	AD141B	AD141C
Median cal. age yrs BP	27593	27593	27593	21885	21885	21885	12265	12265	12265	3492	3492	3492
Total reads	23876	52478	31935	57975	53202	32808	19840	63473	64659	47241	94905	225
Albugo												
Hyaloperonospora									65	1020		2
Pythium												
Pucciniaceae												
Ustilaginomycotina												
Fusarium culmorum												
Phytophthora												
Colletotrichum												
Dothideomycetes				211	1390	159	25	400	341	1840	4614	2

Supplementary Table 2. Radiocarbon ages of rodent middens from the central Atacama and mass of midden subsamples used for DNA extraction.

Radiocarbon age	Error	Median cal. BP	Radiocarbon lab no.	DNA lab no.	Midden code	Mass used in DNA extraction (g)	Locality	Middens site elevation (m above sea level)
200	20	197	UCIAMS 97090	AD 122	CDA-616A	4.967	Cerros de Aiquina	3355
1395	20	1284	UCIAMS 97077	AD 113	CDA-550A	4.023	Cerros de Aiquina	3352
1400	20	1286	UGAMS 8963	AD 137	CDA-507A	5.079	Cerros de Aiquina	3187
2245	20	2231	UCIAMS 97082	AD 111	CDA-595B	4.695	Cerros de Aiquina	3140
2390	25	2358	UGAMS 8966	AD 112	CDA-571	4.862	Cerros de Aiquina	3164
2420	25	2406	UGAMS 8964	AD 129	CDA-545A	4.999	Cerros de Aiquina	3196
3305	42	3492	AA 69837	AD 141	LDT-224	5.919	Lomas de Tilocalar	2872
4475	20	5020	UCIAMS 97081	AD 115	CDA-563B	3.445	Cerros de Aiquina	3173
5920	90	6697	GX 26622	AD 124	CDA-458	5.320	Cerros de Aiquina	3174

6545	25	7424	UCIAMS 97086	AD 132	CDA-561A	5.526	Cerros de Aiquina	3100
6695	25	7532	UCIAMS 97075	AD 120	CDA-575	5.850	Cerros de Aiquina	3111
7325	25	8098	UCIAMS 97078	AD 121	CDA-506A	5.526	Cerros de Aiquina	3189
7325	25	8098	UCIAMS 97088	AD 131	CDA-574A	5.735	Cerros de Aiquina	3115
7600	30	8377	UGAMS 8970	AD 119	CDA-574F	5.777	Cerros de Aiquina	3115
8030	25	8871	UCIAMS 97079	AD 127	CDA-506B	5.218	Cerros de Aiquina	3189
8230	25	9127	UCIAMS 97084	AD 116	CDA-503	4.895	Cerros de Aiquina	3134
8230	70	9156	GX 26621	AD 114	CDA-456	4.372	Cerros de Aiquina	3150
8240	30	9150	UGAMS 8967	AD 133	CDA-595A	6.033	Cerros de Aiquina	3140
8640	30	9547	UGAMS 8969	AD 130	CDA-574C	5.289	Cerros de Aiquina	3115
9230	30	10341	UGAMS 8968	AD 117	CDA-561B	4.841	Cerros de Aiquina	3100
10457	77	12265	AA 69834	AD 140	LDT-210	4.908	Lomas de Tilocalar	2893

10935	30	12752	UCIAMS 97083	AD 125	CDA-186A	5.386	Cerros de Aiquina	3240
11105	35	12927	UCIAMS 97091	AD 123	CDA-502	5.148	Cerros de Aiquina	3108
12795	40	15194	UCIAMS 97085	AD 136	CDA-505	5.181	Cerros de Aiquina	3165
13300	35	15937	UGAMS 8971	AD 128	CDA-535	5.389	Cerros de Aiquina	2967
18100	150	21885	AA 69840	AD 139	LDT-230B1	5.859	Lomas de Tilocalar	2872
23430	?(50)*	27593	AA 65824	AD 138	VDT-204B	5.286	Vegas de Tilocalar	2414
49600	3600	^	UCIAMS 97076	AD 135	CDA-593A	5.84	Cerros de Aiquina	3116

* Error not recorded, 50 used for calibration; ^ outside calibration limits
